# Taking the Path Into the Dark Woods – Initial Industry Impact of the COVID-19 Pandemic

**DOI:** 10.3389/fcimb.2022.841825

**Published:** 2022-07-13

**Authors:** Adam Thornberg

**Affiliations:** GenMark Diagnostics, A Member of the Roche Group, Scientific and Medical Affairs, Carlsbad, CA, United States

**Keywords:** molecular diagnostics, COVID-19, SARS-CoV-2 testing, industry, virology

## Abstract

During the initial onset of the COVID-19 pandemic, each industry experienced hardships. One area that has not been explored in great detail was how the diagnostic industry managed while bringing SARS-CoV-2 tests to the market. This perspective piece provides a sample view of what went on behind the walls of a diagnostic manufacturer that released one of the initial SARS-CoV-2 testing options, some of the barriers that were encountered, and how they could be overcome.

## Perspective

Writing on a topic that needs no introduction can be difficult. It goes without saying that there are plenty of facts that can be strewn about here with regard to COVID-19, like the number of cases, hospitalizations, deaths, etc. Entering year 3 of the pandemic, each individual across the globe has likely had considerable speedbumps in some capacity or another due to the pandemic, provided said individual is in the camp that believes that we are in a global pandemic. The amount of time that has passed since this all began is a bit ludicrous to conceptualize still to this day, but one thing that may be more incredible is the amount of effort that has been deployed worldwide to combat this pandemic, in so many ways, in order to keep people safe.

As we enter the next wave of the Greek alphabet, Omicron and its variants stirring up the most recent excitement (which could easily change by the time this is published), it is prudent to remember that before Omicron, Delta, Alpha and all the others, there was a non-descript pneumonia of unknown origin that caused severe respiratory distress in a patient in China. It seems like almost a lifetime ago, but from the month of December 2019 on, day by day, a virus swept through the human population like wildfire. Spreading from city to city, town to town, country to country and eventually continent to continent. Severe Acute Respiratory Syndrome Coronavirus 2, or SARS-CoV-2, made haste with an impression of impending doom everywhere it surfaced.

There are multiple different vantage points of the COVID-19 pandemic. One that maybe was underrepresented during the earlier days was that of the diagnostic manufacturer. No one can deny what the frontline healthcare professionals were facing. Most of the healthcare industry saw it and even still, it is probably understated. Past the frontline workers, visibility started to dilute out layer by layer, so that by the time it got to the diagnostic manufacturers of SARS-CoV-2 tests, there was not much insight on how the industry was managing to the outside world. This perspective provides a unique view from a diagnostic manufacturer during the initial onset of the pandemic.

Firstly, the decision to make a SARS-CoV-2 test had to be brought to the table. In the beginning, around January 2020, no one really knew which direction the pandemic was going to go. From the perspective of a smaller company (at the time), this leads to one very important decision to make: Do we drop all other projects and allocate resources and funds to develop a very specific niche test that may not have long-term demand or any return on investment? The final answer to this question for GenMark Diagnostics, said smaller company, focusing on multiplex molecular diagnostics for infectious diseases, was yes. The decision was made in January 2020 when the cases were just beginning to make their way outside of China. At that point, it was clear this would be an all hands on deck approach. With only a small number of test manufacturers going down the same very dark road that had very limited visibility, the Research and Development (R&D) team quickly pivoted to researching the SARS-CoV-2 genome to find conservative regions to target for PCR assays. At this point, it is pivotal to remember there were a limited number of sequences available in the GSAID database to work with, alongside minimal knowledge of how well this virus could and would mutate. In fact, there were only about 40 sequences available to be analyzed for assay development purposes. Luckily the CDC-developed test targeting the N gene had given a little runway on what might be a good location to potentially look at.

With this information on hand, R&D located two conserved regions that could be targeted in the N gene, the development could move from in silico analyses to wet benching, followed by manufacturing and into full scale launch. Luckily for GenMark (and likely other manufacturers), a universal cartridge design allows for flexibility in assay development, making it straightforward to place primers and probes on an already FDA-cleared diagnostic test cartridge and platform. Despite this, R&D faced many unforeseen hurdles during the month long development phase. During the initial phases of the pandemic, oligonucleotides were in short supply due to the demand from other companies also embarking on the same journey, but this was far from the only limiting factor. In addition to limited available sequences, commercialized controls were not obtainable to both vendors and laboratories because no manufacturer had started making them yet. Clinical samples were in scarce supply as well and only a few designated labs in the country had them, leading to a very high demand for a very small allocation of samples. This did not distract the team from the goal though. Controls were made through plasmid constructs to confirm primer designs, limit of detection (LoD) studies were completed with *in vitro* transcribed target material and sensitivity and specificity was outsourced to a clinical laboratory that had access to some patient samples. For summation on how quickly the process happened, the clock started on day 1 with checking primers on the bench and kits were shipped out by day 19 for clinical validation. R&D worked tirelessly around the clock and the necessary studies were completed in order to launch as a Research Use Only (RUO) test on March 2, 2020.

R&D achieved a herculean effort in the development of this test and now the next important facets needed to fall into place to mobilize these efforts. In a diagnostic company of any size, there are a lot of individuals that are required to launch a product and generally these people will have ample time for meetings and coordination before a product is brought to market. One department that was heavily leaned on during this time was Regulatory Affairs (RA), comprised of team members that were consistently navigating an ever-changing and sparsely guided regulatory outlook for Emergency Use Authorization (EUA) for a SARS-CoV-2 test. At the time, there was limited guidance on what the FDA was going to accept for a test to be authorized, and the test could not be marketed or sold until authorization came through. The FDA wanted LoD, inclusivity, specificity/cross reactivity and clinical performance. Albeit with limited accessible resources, the RA team diligently researched the needs for submission and kept as in touch as possible with FDA while compiling data from internal and external studies to ensure that all possible aspects dotting i’s and crossing t’s were complete before submission to ensure a speedy review. Once the qualifications for submission were met and the test was submitted for EUA for full review, prior to when the FDA changed their guidance which allowed a test to be marketed and sold prior to authorization if all required components were submitted to FDA. After 8 days on high alert to return any questions to FDA if prompted, on March 19, 2020, the EUA for the GenMark Dx® ePlex® SARS-CoV-2 Test was granted and the test was delivered to the market. However at the time, 8 days felt more like 8 months.

Delivering a test with such rapidity creates its own series of bottlenecks in many other areas of the business. First and foremost, as many will likely recall, that timeframe was during the lockdown in the United States when very little information was available to anyone on how to stop and/or control the spread of COVID-19. This made working in any setting with other people challenging. Manufacturing lines across any industry could be problematic given the proximity of workers for long periods of times. Couple this with a crumbling supply chain, sick time, exposures, and many individuals having to pivot from office to a work-from-home environment, things went wayward quickly. Every manufacturer had to deal with these issues in some capacity, which in turn meant that instruments and test kits could not be manufactured and pushed out the door as fast as they were being sold. The constant question of for labs and manufacturers alike at the time was “How do we scale up?”. It’s hard to scale up in an environment where the world is shutting down and hard to produce more when scaling up rapidly is difficult, which ultimately meant that industry-wide, there were not enough test kits or instruments to meet demand. Noting these challenges, there needed to be a considerable amount of evolution that needed to take place. Internally, companywide policies for health screening (temperature checks, health questionnaires, etc.), masking, special cleaning processes and distancing (including implementation of working from home for non-essential employees) helped to keep cases down internally allowing the manufacturing teams to continue working onsite to provide keep the lines going for product. Over time, additional shifts and positions were opened to try and help bulk up the manufacturing team to meet demands. Externally, teams working from home quickly adapted to Zoom and Microsoft Teams meetings and a more electronically focused work environment, while Supply teams canvased and qualified different manufacturers to see where additional materials could be sourced when necessary.

Customer service roles were also tasked with a different set of responsibilities than usual. Account Representatives along with Customer Service and Product Management had to manage the anticipated supply output and do their best to deliver as many kits or instruments to labs as possible. Being one of the few first tests released, the demand far exceeded the inventory. This created frustration both with laboratories that desperately needed kits and capacity to test them, but also internally when there was not enough material to go around due to the manufacturing and supply chain constraints. All functions had to align daily to make sure that every single kit that was made was shipped immediately under allocation efforts to make sure that all kits got to as many customers as possible. Not to be forgotten, the courageous Molecular Applications Specialists (MAS) were still traveling around the country to install instrumentation and train laboratories not knowing the level of safety around them at any time. The dedication to keep laboratories up and running during this time was undoubtedly a challenge for each person to face every week as they stepped out into a variety of unknowns. This team ensured that customers had their instruments up and running and their staff trained in order to start or keep testing samples for SARS-CoV-2 while also maintaining an outlet for the customer to the company and vice versa, being the only team allowed for in person contact at the time. Without the MAS team, many instruments may have gone uninstalled and a multitude of customers may not have been able to offer our testing solution.

While the in-house Technical Support groups did not have to travel, they had to deal with a different set of challenges like managing limit of detection questions, assay verification to new or unknown comparator methods (in which labs usually had more than 1-2 comparator methods at any given time), ever-changing sample types and discrepant results. By now, it is widely known that many manufacturers took different routes to make a product to detect SARS-CoV-2, but at the time it was very difficult to predict how different chemistries, primer/probe gene locations, number of genes targeted, LoD, sample to answer versus traditional molecular approaches and/or how different primary sample types were going to perform for each company’s assay and all the while, each company is only allowed to support its own assays. Supporting a new product is always a new endeavor for the Technical Support teams but doing it on the fly with so many confounding variables and comparator tests, along with the clinical ramifications for both false negatives and false positives became a new challenge that was not predicted by anyone. The Technical Support groups still assisted customers in any way they could by relying on the instructions for use (IFU) not only for ePlex but other tests/instruments as well, FDA guidance, internally performed studies and engaging different functions within the organization, like R&D, Marketing, MAS’s and Scientific and Medical Affairs, to help provide as much information as possible, while noting that they could only support issues that arose with the ePlex instrument and the ePlex SARS-CoV-2 Test.

The data, experiments, publications, pre-prints and any other outlet of information being released had to be digested on an almost immediate basis by Scientific and Medical Affairs to ensure that any customer had an outlet to ask questions, although many times there was not a clear answer. Even though the data were desperately needed, staying on top of the swirling tornado of data coming in from so many places across the world remained a challenge because it was hard to know what was reliable and what wouldn’t stick, particularly for the pre-prints still pending peer-review. With only two people, the work had to be split evenly to make sure there was efficiency while trying to avoid overlap. Constant data and information exchange was critical as was fully discussing the meaning and outcomes of the research that was surfacing all while also utilizing different working time zones internally and externally to get the most out of each day for GenMark and customers alike.

Bioinformatics had a similar challenge trying to deal with sequences that were constantly mutating and with an extremely limited database of information to help provide definitive clarity on the ability for a test to detect a particular newly emerging strain. The team set up specific cadences to mine and search for new information as it surfaced, passing it on to all internal stakeholders in R&D, Marketing, Scientific and Medical Affairs and Regulatory, while the aforementioned teams would also feed in any information of new strains back to the Bioinformatics team for review and perspective.

All this said, there are so many things that can be elaborated on here and other efforts that can be detailed, but the main point is to highlight the tremendous effort made by the diagnostic community rising to the occasion and releasing high quality testing materials which now stretch across many manufacturers. It was a very difficult and uncertain time early on and given the opportunity, it is relevant to give kudos to the diagnostic industry and all of the internal teams and individuals who made testing possible in the early days in the face of so many challenges and roadblocks. It is key to consider that every function in an organization played a critical role during this pandemic. For some functions like R&D and Scientific and Medical Affairs, all the answers may not always be point blank and may have required scientific intel and fortitude to guide the way while others like Regulatory, Manufacturing and Customer Support teams relied on industry experience and tactile response management to situations as they arose. While it is clear that COVID-19 is not going away anytime soon, it is good to know that there are ways to adequately test for it in so many different capacities now than ever before and hopefully the experiences learned in this round prepare us in case of future rapid onset pandemics.

## Data Availability Statement

The original contributions presented in the study are included in the article/supplementary material. Further inquiries can be directed to the corresponding author.

## Author Contributions

The author confirms being the sole contributor of this work and has approved it for publication.

## Conflict of Interest

AT is employed by Roche Diagnostics.The author declares that the research was conducted in the absence of any commercial or financial relationships that could be construed as a potential conflict of interest.

## Publisher’s Note

All claims expressed in this article are solely those of the authors and do not necessarily represent those of their affiliated organizations, or those of the publisher, the editors and the reviewers. Any product that may be evaluated in this article, or claim that may be made by its manufacturer, is not guaranteed or endorsed by the publisher.

